# Obesity and mortality among older Thais: a four year follow up study

**DOI:** 10.1186/1471-2458-10-604

**Published:** 2010-10-13

**Authors:** Patama Vapattanawong, Wichai Aekplakorn, Uthaithip Rakchanyaban, Pramote Prasartkul, Yawarat Porapakkham

**Affiliations:** 1 Institute for Population and Social Research, Mahidol University, Salaya, Nakhonpathom, Thailand; 2 Department of Community Medicine, Faculty of Medicine, Ramathibodi Hospital, Rama VI Rd., Rajdevi, Bangkok 10400, Thailand; 3 Department of Education, Faculty of Social Sciences and Humanities, Mahidol University, Salaya, Nakhonpathom, Thailand; 4 Setting Priorities using Information on Cost-Effectiveness Project, Ministry of Public Health, Tiwanon Rd., Nonthaburi, Thailand

## Abstract

**Background:**

To assess the association of body mass index with mortality in a population-based setting of older people in Thailand.

**Methods:**

Baseline data from the National Health Examination Survey III (NHES III) conducted in 2004 was linked to death records from vital registration for 2004-2007. Complete information regarding body mass index (BMI) (*n *= 15997) and mortality data were separately analysed by sex. The Cox Proportional Hazard Model was used to test the association between BMI and all-cause mortality controlling for demographic, socioeconomic, and health risk factors.

**Results:**

During a mean follow-up time of 3.8 years (60545.8 person-years), a total of 1575 older persons, (936 men and 639 women) had died. A U-shaped and reverse J-shaped of association between BMI and all-cause mortality were observed in men and women, respectively. However there was no significant increased risk in the higher BMI categories. Compared to those with BMI 18.5-22.9 kg/m^2^, the adjusted hazard ratios (HR) of all-cause mortality for those with BMI <18.5, 23.0-24.9, 25.0-27.4, 27.5-29.9, 30.0-34.9, and ≥35.0 were 1.34 (95% CI, 1.14-1.58), 0.79 (95% CI, 0.65-0.97), 0.81 (95% CI, 0.65-1.00), 0.67 (95% CI, 0.48-0.94), 0.60 (95% CI, 0.35-1.03), and 1.87 (95% CI, 0.77-4.56), respectively, for men, and were 1.29 (95% CI,1.04-1.60), 0.70 (95% CI, 0.55-0.90), 0.79 (95% CI, 0.62-1.01), 0.57 (95% CI, 0.41-0.81), 0.58 (95% CI, 0.39-0.87), and 0.78 (95% CI, 0.38-1.59), respectively, for women.

**Conclusions:**

The results of this study support the obesity paradox phenomenon in older Thai people, especially in women. Improvement in quality of mortality data and further investigation to confirm such association are needed in this population.

## Background

Obesity has become a global public health concern. The World Health Organization (WHO) estimated that there were 400 million obese adults (as measured by body mass index, BMI) worldwide in 2005 and expected to be 700 million in 2025 [1]. The major health consequences associated with obesity include diabetes mellitus (DM), hypertension (HT), dyslipidemia and cardiovascular diseases (CVDs) [2]. As a result, the global burden of disease attributable to obesity amounted to 2.6 million deaths of which ranked the 7^th ^of mortality and the 10^th ^in term of burden of disease due to leading global risk factors [3]. In Thailand, the burden of disease attributable to obesity was 359114 Disability Adjusted Life Years (DALYs) loss in 2000 [4].

In the past two decades, Thailand, a rapidly economic growth country with the advent of an ageing population, the predominant cause of deaths is now changing from infectious diseases to chronic non-communicable diseases including chronic diseases and cancer [5]. Such changes are likely to be attributable to urbanization and changes in lifestyle leading to the increases in cardiovascular risk factors such as hypertension, diabetes and obesity [6-12].

For obesity in Thailand, recent data from a series of Thai National Health Examination Survey (NHES) I-III, 1991-2004 showed that the prevalence of obesity had been increasing in all age groups. In older people, about one-third of Thai adults age ≥60 years were obese (using Asian criteria of BMI ≥ 25 kg/m^2^) [12]. A reverse association between obesity and mortality among older persons had been reported from several studies in the western countries and China [13-23]. The risk of all-cause mortality increased in the low BMI group and decreased in the middle BMI group and increased again in the obesity group especially for BMI ≥ 35 kg/m^2^; however, such relationship had never been investigated in Thai population. To address the question, using mortality data from a national representative population-based data, this study aimed to examine the relationship of body mass index with all-cause mortality in older persons.

## Methods

### Study population and design

This study used data from the National Health Examination Survey (NHES) III, a population-based survey conducted by the Health Systems Research Institute and the Bureau of Policy and Strategy, Ministry of Public Health, Thailand during January 15-April 15, 2004. A three-stage stratified probability sampling was adopted for data collection. Details in sampling method were already mentioned elsewhere [9]. The final representative samples were 39290 individuals of aged 15 and over. For health assessment, people were interviewed using structural questionnaires containing general questions adapted from the health state description questions used in the WHO Multi-Country Survey Study on Health and Responsiveness 2000-2001 [24]. Weight and height were measured using standard techniques [25].

The present study focused on older persons; subjects aged 60 years and older were included in the analysis (n = 19372). The records of the interviewed individuals had been linked using a unique personal identifier (not all records had personal identifiers) to 2004-2007 death records from vital registration, which had per cent completeness greater than 90 [26,27]. According to the personal identifier completion rate, only 17504 from the total number of older persons (90.4%) could be used to link to vital registration. From this linkage, older persons who died from all causes, except accidents and assault (57 cases), and those without information on health risk behaviours were included. The final number of subjects left in this analysis was 15997 or 82.6% of the total number of older persons.

### Measures

All-cause mortality was the outcome variable of interest. Survival time was calculated as the interval between interview date and date of death or until the date of December 31, 2007 if those older persons were still alive.

The main independent variable, body weight, was measured as BMI (weight in kilogram divided by square of height in meter, kg/m^2^). In this study, it was classified into 7 categories according to the recommendation by WHO expert consultation with some modification [28]: < 18.5 kg/m^2^, 18.5-22.9 kg/m^2^, 23.0-24.9 kg/m^2^, 25.0-27.4 kg/m^2^, 27.5-29.9 kg/m^2^, 30.0-34.9 kg/m^2 ^and ≥ 35.0 kg/m^2^. For other independent variables which were taken into account as potential confounding factors were demo-social variables, namely, age, marital status, residential area, and education, and health risk variables, including, diabetes, hypertension, hypercholesterolemia, smoking, fruit and vegetable consumption and physical activity.

For demo-social confounding variables, age was categorized as follows: "60-69", "70-79", and "80+". Marital status was grouped into 3 categories: "single", "married", and "widowed/divorced/separated". For the education variable, the highest level of educational attainment was used and grouped into: "no education", "elementary level", and "secondary level or higher".

For potential health risk confounding variables, diabetes was defined as a person whose fasting blood sugar was ≥ 126 mg/dL or who had previously been diagnosed and was taking hyperglycaemic lowering drugs or insulin [29]. A hypertensive person was defined as a person whose systolic blood pressure ≥ 140 mm Hg and diastolic blood pressure ≥ 90 mm Hg or who had been diagnosed by a physician and was taking antihypertensive drugs [30]. A person having hypercholesterolemia was defined as a person with total blood cholesterol of ≥ 240 mg/dl (5.7 mmol/L) or who was taking hypercholesterolemia-lowering drugs [31]. We classified smoking behaviour of samples into 3 groups: "non-smoker" referred to a person who never smoked, "past smoker" referred to a person who smoked 100 or more cigarettes during his/her lifetime but stop smoking at the time of interview, "current smoker" referred to a person who smoked 100 or more cigarettes during his/her lifetime and was still smoking at the time of the interview. Seven-day recall data regarding fruit and vegetable daily intake were assigned to 2 groups: "less than 5 servings per day", "5 servings or more per day". For physical activities, people were surveyed using the WHO Global Physical Activity Questionnaire and classified as either "active" or "inactive" [32].

### Statistical analysis

In the initial analysis, age-standardized mortality rates by BMI as well as other health risk behaviours were calculated separately for men and women. Then, the association between BMI with all-cause mortality was analysed using the Cox proportional hazard model. In the models, the hazard of death during 4 years of follow-up was a function of BMI and a set of covariates. Three models were created to assess the relationship of BMI and mortality. The first one was unadjusted model or model without controlling by other covariates. The second was an adjusted model where demo-social and health risk variables were added to control for as potential confounding factors. The third model was analysed for samples excluding those who were current smokers or alcohol drinkers. Interaction between BMI and co-morbidity including diabetes or hypertension were tested using a criteria suggesting interaction of P-value <0.02. All analyses were performed with the Stata/SE 10.0 for Windows statistical software package (StataCorp LP, College Station, TX, USA).

This study was approved by the Institute for the Development of Human Research Protection (IHRP), Ministry of Public Health, Thailand.

## Results

### Characteristics of the studied subjects

Among 15997 older persons, 7742 (48.4%) were male and 8255 (51.6%) were female. According to BMI, 16.3% of male and 13.6% of female had BMI less than 18.5 kg/m^2^, 43.8% of male and 34.0% of female had BMI between 18.5 to 22.9 kg/m^2^, 17.5% of both male and female had BMI between 23.0 to 24.9 kg/m^2^, 13.8% of male and 17.0% of female had BMI between 25.0 to 27.4 kg/m^2^, 5.8% of male and 9.5% of female had BMI between 27.5 to 29.9 kg/m^2^, 2.6% of male and 7.0% of female had BMI between 30.0 to 34.9 kg/m^2^. There were only 0.3% of older men and 1.3% of older women whose BMI were 35.0 kg/m^2 ^or more. Distributions of several factors including demo-social and health risk factors were shown in Table 1. Older people, both male and female, in the higher BMI categories were likely to have younger age, more co-morbidity of diabetes, hypertension, hypercholesterolemia, less smokers, more physically inactive. Those with higher BMI were also had higher education level and resided in urban area.

**Table 1:** Baseline characteristics of studied samples by body mass index (BMI)

Characteristics	BMI (kg/m^2^)							p-value
		
	<18.5	18.5-22.9	23.0-24.9	25.0-27.4	27.5-29.9	30.0-34.9	≥35.0	
MALE (n = 7742)	(n = 1260)	(n = 3392)	(n = 1352)	(n = 1069)	(n = 447)	(n = 200)	(n = 22)	
Age, mean (SD)	70.9 ± 6.7	69.3 ± 6.4	68.5 ± 6.1	67.9 ± 5.8	67.4 ± 5.4	67.1 ± 5.4	65.6 ± 5.4	<.001
Diabetes (%)	6.5	10.3	16.6	22.4	29.3	22.0	40.9	<.001
Hypertension (%)	41.9	48.7	59.3	65.0	68.2	76.5	77.3	<.001
Hypercholesterolemia (%)	9.8	17.3	29.0	31.9	31.8	28.0	36.4	<.001
Smoking (%)								<.001
Non-smoker	22.8	29.9	35.3	41.5	45.2	46.5	54.5	
Past smoker	25.8	28.3	36.0	35.1	37.8	38.0	27.3	
Current smoker	51.4	41.8	28.7	23.4	17.0	15.5	18.2	
Fruit & vegetables consumption per day (%)								<.001
<5 servings	88.0	87.0	83.4	84.1	81.9	82.5	68.2	
> = 5 servings	12.0	13.0	16.5	15.9	18.1	17.5	31.8	
Physical inactive (%)	34.5	30.2	30.4	31.6	36.7	37.5	36.4	<.01
Education level (%)								<.001
No education	14.7	11.0	8.2	7.6	4.9	7.0	18.2	
Elementary	79.9	77.5	73.0	70.3	70.9	69.0	54.5	
Secondary or higher	5.4	11.5	18.8	22.2	24.2	24.0	27.3	
Urban (%)	39.4	46.5	55.8	61.0	62.0	62.0	68.2	<.001
FEMALE (n = 8255)	(n = 1124)	(n = 2807)	(n = 1445)	(n = 1407)	(n = 788)	(n = 575)	(n = 109)	
Age (mean)	71.2 ± 6.9	69.6 ± 6.4	68.4 ± 6.0	68.1 ± 6.0	67.0 ± 5.5	66.9 ± 5.2	67.5 ± 6.0	<.001
Diabetes (%)	6.5	15.2	21.5	23.0	27.4	25.0	23.9	<.001
Hypertension (%)	40.0	47.9	55.5	61.5	64.7	69.6	74.3	<.001
Hypercholesterolemia (%)	16.4	31.5	38.5	43.1	48.7	45.4	45.9	<.001
Smoking (%)								<.001
Non-smoker	80.5	88.0	91.7	93.2	95.2	94.1	94.5	
Past smoker	8.1	5.0	4.5	3.8	3.1	4.2	3.7	
Current smoker	11.4	7.1	3.8	3.0	1.8	1.7	1.8	
Fruit & vegetables consumption per day (%)								<.001
<5 servings	89.3	85.5	83.7	82.7	77.4	78.6	81.7	
> = 5 servings	10.7	14.5	16.3	17.4	22.6	21.4	18.4	
Physical inactive (%)	41.8	39.6	38.1	40.0	39.1	40.9	43.1	>.05
Education level (%)								<.001
No education	32.4	24.6	21.1	18.2	17.3	17.9	13.8	
Elementary	65.8	70.8	71.6	74.7	73.9	76.5	78.9	
Secondary or higher	1.9	4.6	7.3	7.1	8.9	5.6	7.4	
Urban (%)	40.3	47.3	52.9	59.0	61.3	62.6	61.5	<.001

### Age-adjusted all-cause mortality rate by BMI and other potential health risks

During the mean follow-up time of 3.8 years (60545.7 person-years), 1575 subjects (936 men and 639 women) died (9.9%). Median survival time among those who died was 2.25 years (95% confidence interval [CI], 2.12-2.37) and 2.31 years (95% CI, 2.14-2.43) for men and women respectively.

Table 2, the age-adjusted all-cause mortality rates by each BMI category (<18.5, 18.5-22.9, 23.0-24.9, 25.0-27.4, 27.5-29.9, 30.0-34.9, and ≥35.0) for older men were 43.4, 32.4, 26.4, 29.7, 23.9, 22.3, and 64.8 per 1000 person-years, respectively and for older women were 27.1, 22.1, 16.5, 20.1, 14.3, 14.4, and 21.0 per 1000 person-years, respectively. The mortality rates among men were slightly higher than among women for all categories of BMI and a concave pattern was observed in both older men and women. Mortality rates of both older men and women having diabetes or hypertension were higher than those without the conditions except for those who having hypercholesterolemia. Among 3 categories of smoking variable (non-smoker, past smoker, current smoker), the age-adjusted mortality rate of those who smoked in the past was highest while of those who never smoked was lowest. These patterns were observed in both older men and women and, again, older men had higher mortality than older women. Furthermore, either older people who consumed fruit and vegetable less than 5 servings per day or who were physically inactive had higher mortality rates compared to their counterpart.

**Table 2:** Age-adjusted all-cause mortality rate (per 1000 person-years) by health risk characteristics and sex among older Thais

Health risk characteristics	Males			Females		
	
	Person-year	Deaths	**Mortality Rate**^**a **^**(95% CI)**	Person-year	Deaths	**Mortality Rate**^**a **^**(95% CI)**
BMI (kg/m^2^)						
< 18.5	4567.1	222	43.4 (38.1-48.7)	4201.6	134	27.1 (22.9-31.3)
18.5-22.9	13000.0	418	32.4 (29.5-35.2)	10674.3	245	22.1 (19.6-24.6)
23.0-24.9	5112.0	132	26.4 (22.5-30.3)	5586.6	90	16.5 (13.6-19.4)
25.0-27.4	4057.2	107	29.7 (24.8-34.6)	5400.0	95	20.1 (16.6-23.6)
27.5-29.9	1716.4	38	23.9 (17.9-29.8)	3048.2	39	14.3 (10.8-17.9)
30.0-34.9	758.6	14	22.3 (14.2-30.3)	2229.8	28	14.4 (10.3-18.4)
≥ 35.0	78.1	5	64.8 (31.7-97.9)	423.0	8	21.0 (12.2-29.8)
Diabetes						
No	25008.4	764	30.2 (28.1-32.2)	25867.1	466	17.7 (16.2-19.2)
Yes	3973.6	172	45.7 (39.6-51.7)	5696.6	173	32.8 (28.4-37.2)
Hypertension						
No	13519.0	380	28.9 (26.2-31.6)	14613.0	244	17.4 (15.5-19.4)
Yes	15463.1	556	35.2 (32.5-37.9)	16950.6	395	22.7 (20.6-24.7)
Hypercholesterolemia						
No	22795.5	745	32.2 (30.0-34.3)	20360.3	426	20.4 (18.6-22.2)
Yes	6186.6	191	32.1 (28.1-36.2)	11203.4	213	19.9 (17.5-22.3)
Smoking						
Non-smoker	9558.7	262	27.1 (24.2-30.1)	28353.3	552	19.6 (18.0-21.1)
Past smoker	8872.8	327	35.1 (31.6-38.6)	1508.3	44	27.2 (20.8-33.6)
Current smoker	10550.6	347	34.1 (30.8-37.4)	1702.1	43	26.1 (20.0-32.2)
Fruit & vegetables consumption per day						
<5 servings	24785.4	821	32.8 (30.7-34.9)	26454.9	561	21.0 (19.4-22.6)
> = 5 servings	4196.7	115	29.2 (24.6-33.8)	5108.7	78	16.7 (13.5-19.8)
Physical activities						
Inactive	9043.3	363	37.7 (34.1-41.3)	12445.8	325	24.2 (21.8-26.6)
Active	19938.7	573	29.6 (27.4-31.9)	19117.8	314	17.5 (15.8-19.3)

^a ^Standardized by total older persons age structure.

### Association of BMI and all-cause mortality

**Figure 1:**
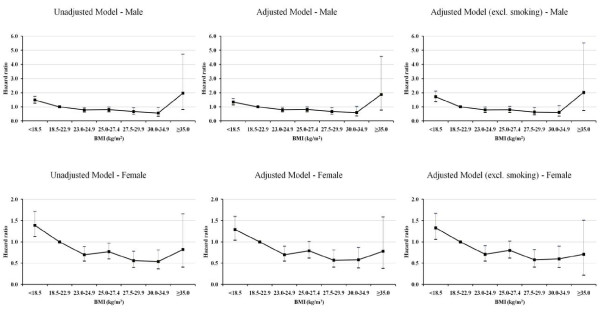
**Hazard ratios and 95% CI by BMI categories**. Adjusted model: controlling for marital status, urban/rural, education, living arrangement, diabetes, hypertension, smoking, and physical activity.

In Cox proportional hazard models (Table 3), there was evidence of the association between BMI and all-cause mortality. A flat U-shaped associations with the highest risk of death in the lowest and the highest categories of BMI (<18.5 kg/m^2 ^and ≥ 35.0 kg/m^2^, respectively) was observed in older men (Figure 1, upper panel) while a reverse J-shaped association with the highest risk of dying in the lowest BMI category (<18.5 kg/m^2^) was observed in older women (Figure 1, lower panel). Among older men, BMI of <18.5 kg/m^2 ^was strongly associated with increasing risk of death but BMI of ≥35.0 kg/m^2 ^was weakly associated with increasing risk of death. Conversely, the other BMI categories were strongly associated with reducing risk of dying in older men. Among older women, the only BMI of <18.5 kg/m^2 ^was strongly associated with increasing risk of death. The higher BMI groups were strongly associated with reducing risk of dying except for the highest BMI category (≥35.0 kg/m^2^).

**Table 3:** Cox Proportional Hazard Ratios and 95% CI (in parenthesis) for survival time among older Thais in NHES III after 4 years of follow-up, 2004-2007

		Males			Females	
	
Variables	Unadjusted Model	**Adjusted Model**^**a**^	**Adjusted Model with Excluding Current Smoking & drinking**^**a**^	Unadjusted Model	**Adjusted Model**^**a**^	**Adjusted Model with Excluding Current Smoking & drinking**^**a**^
	(n = 7742)	(n = 7742)	(n = 4928)	(n = 8255)	(n = 8255)	(n = 7805)
Age (yr) in 2004 (Ref: 60-69)						
70-79	1.87 (1.62-2.16)	1.69 (1.46-1.95)	1.61 (1.33-1.93)	2.07 (1.74-2.46)	1.82 (1.52-2.18)	1.89 (1.57-2.27)
80+	4.31 (3.58-5.19)	3.42 (2.79-4.18)	3.61 (2.83-4.62)	5.07 (4.07-6.33)	3.95 (3.09-5.06)	4.06 (3.15-5.23)
BMI (kg/m^2^) (Ref: 18.5-22.9)						
< 18.5	1.48 (1.26-1.74)	1.34 (1.14-1.58)	1.71 (1.37-2.11)	1.39 (1.13-1.72)	1.29 (1.04-1.60)	1.33 (1.06-1.67)
23.0-24.9	0.78 (0.64-0.95)	0.79 (0.65-0.97)	0.78 (0.61-0.99)	0.70 (0.55-0.89)	0.70 (0.55-0,90)	0.71 (0.55-0.91)
25.0-27.4	0.80 (0.65-0.99)	0.81 (0.65-1.00)	0.79 (0.61-1.03)	0.77 (0.60-0.97)	0.79 (0.62-1.01)	0.80 (0.62-1.02)
27.5-29.9	0.67 (0.48-0.93)	0.67 (0.48-0.94)	0.63 (0.43-0.94)	0.56 (0.40-0.78)	0.57 (0.41-0.81)	0.58 (0.41-0.82)
30.0-34.9	0.56 (0.33-0.95)	0.60 (0.35-1.03)	0.61 (0.34-1.09)	0.54 (0.37-0.81)	0.58 (0.39-0.87)	0.60 (0.40-0.90)
≥ 35.0	1.96 (0.81-4.73)	1.87 (0.77-4.56)	2.02 (0.74-5.52)	0.82 (0.41-1.66)	0.78 (0.38-1.59)	0.71 (0.22-1.51)

^a ^Adjusted for marital status, urban/rural, education, living arrangement, diabetes, hypertension, smoking, and physical activity.

There was a slightly reduction in risk of dying after controlled by all covariates (adjusted model) compared to the unadjusted model. In this model, an interaction between health risk variables, such as BMI with diabetes or BMI with hypertension, as well as an interaction between demo-social variables, such as BMI with age or BMI with sex or BMI with education were tested, but no statistical significance at P-value <0.2 were found among those pairs. In adjusted model, the hazard ratio (HR) according to BMI <18.5 kg/m^2^, 23.0-24.9 kg/m^2^, 25.0-27.4 kg/m^2^, 27.5-29.9 kg/m^2^, 30.0-34.9 kg/m^2^, and ≥35.0 kg/m^2 ^relatively to BMI 18.5-22.9 kg/m^2 ^was 1.34 (95% CI, 1.14-1.58), 0.79 (95% CI, 0.65-0.97), 0.81 (95% CI, 0.65-1.00), 0.67 (95% CI, 0.48-0.94), 0.60 (95% CI, 0.35-1.03), and 1.87 (95% CI, 0.77-4.56), respectively, for men, and was 1.29 (95% CI,1.04-1.60), 0.70 (95% CI, 0.55-0.90), 0.79 (95% CI, 0.62-1.01), 0.57 (95% CI, 0.41-0.81), 0.58 (95% CI, 0.39-0.87), and 0.78 (95% CI, 0.38-1.59), respectively, for women.

Additional analysis was done for model excluding those who were current smoking or drinking (36.3% of total men, 5.5% of total women). In this reducing model, the pattern of association of BMI with all-cause mortality controlling for other covariates did not substantially change.

## Discussion

This is the first population-based and prospective study on BMI and survival among the older people in Thailand. This study takes an advantage of the linkages between two potential sources of data, NHES III and vital registration. The results show evidences of higher mortality rates in people who are underweight, compared to normal weight persons with a higher magnitude in men compared to women. Being overweight or obese appears to have different effects by gender as lower risk of mortality were observed in women but not in men.

This study observes the U-shaped and reverse J-shaped association of BMI with all-cause mortality in older persons as found in many studies [33-36]; however, there is a no significant association in the highest BMI category due to the small number of subjects with BMI ≥ 35.0 kg/m^2 ^(0.3% for men, 1.3% for women). The finding of BMI and mortality is consistent with several studies in the West and Asia [17,20-23,33-35]. Such reverse relationship has been termed as a phenomenon of obesity paradox [37-39]. Some explanations for a lower not a higher mortality risk among the higher BMI older persons includes 'survival effect' where obese persons may have already died from some complications of obesity, leaving only those who are can tolerate [37]. Some older persons with low BMI may have unrecognised illnesses which lead to their higher mortality rates [37]. Some degree of differences in the magnitude of association by gender is found. This may reflect that the obesity paradox is more pronounce in the older Thai women than in men. This issue merits further investigated in this population.

The strength of present study is that it covers a nationwide population with a relatively large sample size. However, there are some limitations that may affect the results of the study. Firstly, this study is focusing on the association of BMI with only all-cause mortality. We are confident on the completeness of the total number of deaths from the registration. However, the quality of registration data on specific causes of death is still in question. Therefore, we are not able to determine the association between BMI and specific causes of death such as CVDs as found in several studies [38,40,41]. Secondly, about 27% of the observations from NHES III are incomplete. However, it is found from our cross-tabulation (data not shown) that the characteristics of those cases with incomplete data are similar to those included in the analysis. Thirdly, information on co-morbidity of chronic diseases was not available and this might overestimate the mortality risk in the lower BMI category. Fourthly, quality of life might be related to obesity and the decreased in physical function in older persons, as well as mortality; however, quality of life had not been properly measured in this study, so this issue was not included. Lastly, the follow-up time of 4 years might not be long enough to cover the effects of obesity. A long term follow-up of this cohort might be warrant to confirm the association of BMI with mortality among older Thai people. Effort to investigate the cause of death could be encouraged in order to enhance the findings of association between BMI and specific cause of death.

## Conclusions

The results of this study support the obesity paradox phenomenon in older Thai people, especially in women. It shows that being underweight is a strong predictor of mortality in both men and women, while being obese is a protective factor of mortality in the older women. Improvement in quality of mortality data and further investigation to confirm such association are needed in this population.

## Competing interests

The authors declare that they have no competing interests.

## Authors' contributions

PV compiled the data, undertook the analysis, and drafted the initial article. WA provided technical advice, contributed to draft revisions and the coordination of the research. UR contributed to analysis and interpretation of results. PP provided technical advice and contributed to the discussion. YP generated the initial idea of the study and contributed to drafted revisions. All authors agreed on the manuscript.

## Pre-publication history

The pre-publication history for this paper can be accessed here:

http://www.biomedcentral.com/1471-2458/10/604/prepub
